# Supplementary material for: Behaviour and reproduction of Drosophila melanogaster exposed to 3.6 GHz radio-frequency electromagnetic fields
Source: PLoS One. 2025 Dec 1;20(12):e0336228. doi: 10.1371/journal.pone.0336228 (PMC12668527; doi:10.1371/journal.pone.0336228)
Supplement: S1 File — (DOCX) [file pone.0336228.s001.docx]

**Extended Methods**

***Micro-CT Scanning of a Drosophila Melanogaster Fly***

The X-ray tube was operated at 80 kVp and 36 μA, delivering a power of 2.88 W. The sample was positioned at a source-to-object distance of 10.13 mm and a source-to-detector distance of 978.73 mm, resulting in a geometric magnification factor of 96.7x. Each projection was acquired using 4-frame averaging with an exposure time of 500 ms per frame. The detector, binned 2x to a resolution of 1425 × 1425 pixels, provided an isotropic voxel size of 3.1 μm. Reconstructed data were then processed using a 3x3x3 median filter to improve signal-to-noise ratio and saved as a 16-bit tiff-stack.

***Dosimetry of Fecundity Experiments***

The fecundity experiments took place in the near field of an emitting antenna. In our dosimetric assessment, we took the following steps: the source was modeled as a dipole antenna resonating at 3.6 GHz, emulating the stub antenna used during the experiment (see Fig. 3(c)). The dipole consisted of two perfectly conducting cylinders with length 166 mm and radius 0.378 mm, separated by a voltage source of 7.2 mm. The dipole has an |S11|² of -14.86 dB at 3.6 GHz, which indicates that 96.73 % of the power is accepted into the dipole. The input power into the dipole was 23.5 dBm in the simulation, which corresponded to a power of 1.32 nW at 30 cm distance from the dipole, in good agreement with the value of 1.29 nW that was measured in the experimental configuration. Mann-Whitney U testing on measurements with and without vials could not prove that the vials attenuate the electromagnetic field. Therefore, the vials were not considered in the near-field simulations. The food within the vials was also not included in the simulations. As the flies moved freely within the vials during the experiments, the near field simulations were executed for 12 fly locations, each with two orientations as shown in Figure S1. We assume that the overall absorbed dose of the flies during the experiments can be estimated by averaging the doses at these locations. Due to the radial symmetry of the antenna the doses at locations 1 and 2, 5 and 6, 9 and 10 (see Fig. S1) can be considered equal. In all near field simulations, the grid step was set at 0.3 µm and the number of simulated periods was 20.

***Characterization of RF-EMF Exposure Inside the Incubator***

During the measurements, the RF-EMF emitting antenna was in the same location as during the behavioral experiments, and a Narda NBM Probe with EF 0691 E-Field probe (Narda, Hauppage, NY) was used to register root-mean-square electric field strength 100 kHz- 6 GHz every 2 seconds. Simultaneously, the antenna was fed the same 15 dBm of RF power at 3.6 GHz it was fed during the experiments. We measured 3 E_RMS_ values (in V/m) on 3 locations in the incubator: ‘Bottom’, corresponding to the bottom of the locomotion device (device removed), ‘top’, corresponding to the top of the device (device present), and ‘antenna’, with the RF-probe at 1 cm from the emitting antenna (device present). The same measurements were repeated with the antenna disconnected from the RF probe (sham exposure). E_RMS_ values were collected in each condition, summary statistics were calculated and the Mann-Whitney U test was used to test whether E_RMS_ depended on distance and the RF-EMF antenna emitting or not.

Our assumption was that most of the E-field inside of the incubator was caused by the 3.6 GHz emissions and that the metal incubator shields environmental RF-EMFs. However, the Narda broadband probe only provides amplitude information and no frequency information. Therefore, we also performed measurements with an ExpoM-RF 3 (Fields at Work, Zürich, Switzerland), which does give frequency-specific information. The ExpoM was placed at the same location where the locomotion registering device was placed and registered RF-EMF values in 16 common telecom bands covering 100 – 6 GHz, including the 3.5-3.7 GHz telecom band, in which we were emitting. The device registered E_RMS_ in each of these bands every 3 s during one hour with the RF source off (sham) and emitting 3.6 GHz at 15 dBm. This led to 1190 E_RMS_ samples measured in each frequency band. These were in each time-instance summed quadratically over all frequency bands in order to obtain, by taking a square root of this sum, the total E-field strength E_RMS,tot_ for which summary statistics were calculated in each condition and which was compared to E_RMS_,_3.6GHz_ the E-field strength at 3.6 GHz, again using MWU testing. Additionally, we conducted background measurements of RF exposure in the lab where the fecundity tests occurred, using the ExpoM-RF3 to derive mean total and 3.6 GHz-specific E-field strengths.

***Characterization of RF-EMF Exposure: Fecundity Experiments***

For the fecundity experiments, we used the RF Explorer Probe (Seeed Studio) to measure the received power on the probe at 3 different distances from the collection of vials: at 1 cm, 10 cm, and 30 cm from the vials with the RF-EMF source emitting 3.6 GHz at 15 dBm and turned off. The same measurements were also repeated without the vials being present to quantify the loss inside of the vials. Additionally, we also performed measurements at 1 cm from the emitting monopole without vials being present. At each measurement position, we collected 30 samples, which were used to calculate summary statistics, to test for a distance dependency, and to test a difference between the conditions: RF ON versus RF OFF and with versus without vials.
