# Supplementary material for: Behaviour and reproduction of Drosophila melanogaster exposed to 3.6 GHz radio-frequency electromagnetic fields
Source: PLoS One. 2025 Dec 1;20(12):e0336228. doi: 10.1371/journal.pone.0336228 (PMC12668527; doi:10.1371/journal.pone.0336228)
Supplement: S2 File — (DOCX) [file pone.0336228.s002.docx]

**Extended Results**

***Characterization of RF-EMF Exposure Inside the Incubator***

Table S2 lists the measured E-field strength in the incubator with and without an RF source connected. It is clear by comparing the average values that the dominant source of RF exposure within the incubator is the RF-emitting antenna, with average E-field levels approximately a factor 100 times higher with RF and without RF. Statistical testing confirmed this with MWU tests returning p values lower than 0.01 in all pairwise tests comparing RF ON and OFF.

With RF OFF, all average exposure values are below 0.1 V/m, indicating that the incubator potentially shields all external RF fields or that the environmental level is at this value. With RF ON, we find that the fields are higher near the antenna than at the location of the experiment further from the antenna (MWU, p<0.01). This is expected since RF fields should in general decay as they propagate further from the RF source. At the bottom, we find a higher total exposure than 3.6 GHz exposure, even though our RF source emits a pure sine wave at 3.6 GHz. We attribute this difference to cross-talk, a misclassification of E-fields in neighbouring frequency bands [Thielens et al., 2014]. This is confirmed by our measurements where the adjacent WiFi 5GHz frequency band shows *3.7*$\pm0.3 V/m$ with the RF ON and *0.02*$\pm0.0.008 V/m$ with RF OFF*,* while there was no change in the use of the WiFi network or no WiFi emitting or receiving devices near or in the incubator. We executed a measurement of background environmental RF-EMF field strengths in the lab, by placing the ExpoM-RF3 on top of the incubator for 10 minutes with a 3 s sampling rate, which resulted in an E-field strength of 0.035+/-0.019 V/m in total and 0.0014 +/- 0.0001 V/m at 3.6 GHz. While these results are executed over a timespan that is too short to give a general overview of the RF-EMF exposure outside of the incubator, they show that the environmental exposure was relatively low and that there was no strong active 3.6 GHz emitter in the lab that could confound the experiments. In summary, we show that our exposed condition receives a significantly higher RF exposure than the control condition and that this exposure is at 3.6 GHz.

***Characterization of RF-EMF Exposure: Fecundity Experiments***

Table S3 lists the RF power (W) measured by the RF Explorer (Seeed Studio) at several distances from the monopole antenna during the fecundity experiments. The RF OFF measurements differ considerably from RF ON measurements, which is confirmed by MWU tests with p-values smaller than 0.01. The vials seem to have a negligible attenuating effect. MWU testing could not prove that the values stem from a different distribution (p-values 0.21, 0.17 and 0.48 for distances 1 cm, 10 cm and 30 cm from the antenna resp.). Because no direct electric field strength measurements were performed during the fecundity experiments, the stub antenna is modelled as a dipole antenna in Sim4life. The dipole antenna was designed to approximate the power measurements at 30 cm from the vials and, as can be seen in Table S3, is in good agreement with the values at the other 3 distances as well.
