# Supplementary material for: Behaviour and reproduction of Drosophila melanogaster exposed to 3.6 GHz radio-frequency electromagnetic fields
Source: PLoS One. 2025 Dec 1;20(12):e0336228. doi: 10.1371/journal.pone.0336228 (PMC12668527; doi:10.1371/journal.pone.0336228)
Supplement: S1 Table — (DOCX) [file pone.0336228.s003.docx]

**S1 Table. Dielectric properties of insect tissues at 1-240 GHz as obtained in [Thielens et al., 2018]**

|  | 1 GHz | 2.45 GHz | 3.6 GHz | 6 GHz | 12 GHz | 26 GHz | 60 GHz | 90 GHz | 120 GHz | 240 GHz |
| --- | --- | --- | --- | --- | --- | --- | --- | --- | --- | --- |
| Relative permittivity | 48.6 | 43.9 | 42 | 38.2 | 27 | 15.4 | 7.3 | 6.1 | 5.5 | 5.1 |
| Conductivity (S/m) | 1.2 | 1.9 | 2.8 | 5.2 | 10.7 | 20.8 | 27.1 | 28.4 | 28.8 | 29.5 |
