# Supplementary material for: Behaviour and reproduction of Drosophila melanogaster exposed to 3.6 GHz radio-frequency electromagnetic fields
Source: PLoS One. 2025 Dec 1;20(12):e0336228. doi: 10.1371/journal.pone.0336228 (PMC12668527; doi:10.1371/journal.pone.0336228)
Supplement: S2 Table — Columns indicate different conditions and measurement devices, while the rows indicate different measurement locations. (DOCX) [file pone.0336228.s004.docx]

**S2 Table. Measured RF-EMF Electric Field strengths (in V/m) corresponding to the behavioural experiments. Columns indicate different conditions and measurement devices, while the rows indicate different measurement locations.**

|  | *3.6 GHz, 15 dBm* | | | *RF OFF* | | |
| --- | --- | --- | --- | --- | --- | --- |
|  | *E_RMS,tot*  *Narda^a^* | *E_RMS,tot*  *EXPOM^b^* | *ERMS,3.6*  *EXPOM^b^* | *E_RMS,tot*  *Narda^a^* | *E_RMS,tot*  *EXPOM^c^* | *ERMS,3.6*  *EXPOM^c^* |
| *Antenna* | $19\pm2.4 V/m$ | *-* | *-* | *0.037*$\pm$ *0.048*$V/m$ | *-* | *-* |
| *Top* | $7.6\pm1.4 V/m$ | *-* | *-* | *0.09* $\pm$*0.07*$V/m$ | *-* | *-* |
| *Bottom* | *9.0*$\pm0.7 V/m$ | *6.7*$\pm0.5 V/m$ | $5.6\pm0.4 V/m$ | *0.067*$\pm$ *0.060*$V/m$ | *0.034*$\pm$ *0.021*$V/m$ | *0.006*$\pm$ *0.004*$V/m$ |
| *^a^N=30, ^b^N=1193, ^c^N=1163* | | | | | | |
