# Supplementary material for: Behaviour and reproduction of Drosophila melanogaster exposed to 3.6 GHz radio-frequency electromagnetic fields
Source: PLoS One. 2025 Dec 1;20(12):e0336228. doi: 10.1371/journal.pone.0336228 (PMC12668527; doi:10.1371/journal.pone.0336228)
Supplement: S3 Table — Columns indicate different conditions, while the rows indicate different measurement locations. The measurements were carried out with the RF Explorer (Seeed Studio) in N = 30 repetitions. (DOCX) [file pone.0336228.s005.docx]

**S3 Table. Measured RF-EMF power corresponding to the fecundity experiments. Columns indicate different conditions, while the rows indicate different measurement locations. The measurements were carried out with the RF Explorer (Seeed Studio) in N=30 repetitions.**

|  | *RF OFF* | *3.6 GHz, 15 dBm* | | *Simulated dipole antenna* |
| --- | --- | --- | --- | --- |
|  | *Without vials* | *With vials* | *Without vials* | *Without vials* |
| *1 cm from antenna* | *1.69E-14* $\pm$ *4.33E-15*$W$ | *-* | *10.3*$\pm$ *1.41*$mW$ | *12.6 mW* |
| *1 cm from vials* | *1.59E-14* $\pm$ *2.13E-15*$W$ | *5.18*$\pm$*0.479 mW* | *5.29*$\pm$ *0.475*$mW$ | *4.25 mW* |
| *10 cm from vials* | *1.51E-14*$\pm$ *1.80E-15*$W$ | *0.754*$\pm$ *0.0513*$mW$ | *0.761*$\pm$ *0.0452*$mW$ | *0.951 mW* |
| *30 cm from vials* | *1.81E-14*$\pm$*5.18E-15 W* | *1.29*$\pm$ *0.413*$nW$ | *1.37* $\pm$ *0.422*$nW$ | *1.32 nW* |
