# Supplementary material for: Behaviour and reproduction of Drosophila melanogaster exposed to 3.6 GHz radio-frequency electromagnetic fields
Source: PLoS One. 2025 Dec 1;20(12):e0336228. doi: 10.1371/journal.pone.0336228 (PMC12668527; doi:10.1371/journal.pone.0336228)
Supplement: S4 Table — (DOCX) [file pone.0336228.s006.docx]

**S4 Table. Absorbed power (nW) in D. melanogaster tissues from 1 V/m far-field exposure at 3.6 GHz for 12 polarizations (E1 to E12).**

|  | Internal tissue | Exoskeleton | Brain | Halteres | Wings | Gonads | Muscles | Total |
| --- | --- | --- | --- | --- | --- | --- | --- | --- |
| Volume | 1.9 mm^3^ | 0.072  mm^3^ | 0.059 mm^3^ | 0.0012 mm^3^ | 0.039 mm^3^ | 0.0017 mm^3^ | 0.067 mm^3^ | 2.1 mm^3^ |
| E1 | 0.0349 | 0.00576 | 3.92E-4 | 4.04E-5 | 0.00891 | 5.37E-6 | 3.14E-4 | 0.0503 |
| E2 | 0.191 | 0.0122 | 0.00630 | 6.04E-5 | 0.0207 | 8.46E-5 | 0.00601 | 0.236 |
| E3 | 0.0597 | 0.00869 | 3.59E-4 | 2.05E-5 | 0.00522 | 2.48E-5 | 7.43E-4 | 0.0748 |
| E4 | 0.186 | 0.0231 | 0.00623 | 8.08E-5 | 0.0162 | 9.11E-5 | 0.00604 | 0.238 |
| E5 | 0.0600 | 0.00871 | 3.61E-4 | 1.99E-5 | 0.00521 | 2.35E-5 | 7.47E-4 | 0.0750 |
| E6 | 0.0327 | 0.0107 | 3.91E-4 | 7.53E-5 | 0.00723 | 5.29E-6 | 3.25E-4 | 0.0514 |
| E7 | 0.0564 | 0.0159 | 3.65E-4 | 3.81E-5 | 0.00445 | 2.82E-5 | 7.61E-4 | 0.0779 |
| E8 | 0.0326 | 0.0107 | 3.95E-4 | 7.79E-5 | 0.00724 | 6.52E-6 | 3.19E-4 | 0.0514 |
| E9 | 0.186 | 0.0231 | 0.00615 | 8.15E-5 | 0.0162 | 9.30E-5 | 0.00605 | 0.238 |
| E10 | 0.0326 | 0.0107 | 3.80E-4 | 7.58E-5 | 0.00723 | 5.66E-6 | 3.20E-4 | 0.0512 |
| E11 | 0.186 | 0.0231 | 0.00619 | 7.98E-5 | 0.0162 | 9.10E-5 | 0.00604 | 0.238 |
| E12 | 0.0561 | 0.0159 | 3.70E-4 | 3.69E-5 | 0.00445 | 2.62E-5 | 7.54E-4 | 0.0776 |
| Mean | 0.0928 | 0.0140 | 0.00232 | 5.73E-5 | 0.00994 | 4.04E-5 | 0.00237 | 0.122 |
