# Supplementary material for: Behaviour and reproduction of Drosophila melanogaster exposed to 3.6 GHz radio-frequency electromagnetic fields
Source: PLoS One. 2025 Dec 1;20(12):e0336228. doi: 10.1371/journal.pone.0336228 (PMC12668527; doi:10.1371/journal.pone.0336228)
Supplement: S5 Table — These were obtained by rescaling the total absorbed power that was computed in the simulations by the squared electric field strength in the behavioural experiments. (DOCX) [file pone.0336228.s007.docx]

**S5 Table. Absorbed power (nW) in D. melanogaster tissue during the behavioral experiments under the assumption of a lower bound and upper bound electric field strength of 5.4 and 9 V/m resp. These were obtained by rescaling the total absorbed power that was computed in the simulations by the squared electric field strength in the behavioural experiments.**

| Polarization | Absorbed power (nW) |
| --- | --- |
| E1 | 1.47 – 4.07 |
| E2 | 6.88 – 19.1 |
| E3 | 2.18 – 6.06 |
| E4 | 6.94 – 19.3 |
| E5 | 2.19 – 6.08 |
| E6 | 1.50 – 4.16 |
| E7 | 2.27 – 6.31 |
| E8 | 1.50 – 4.16 |
| E9 | 6.94 – 19.3 |
| E10 | 1.49 – 4.15 |
| E11 | 6.94 – 19.3 |
| E12 | 2.26 – 6.29 |
| Mean | 3.56 – 9.88 |
