# Supplementary material for: Behaviour and reproduction of Drosophila melanogaster exposed to 3.6 GHz radio-frequency electromagnetic fields
Source: PLoS One. 2025 Dec 1;20(12):e0336228. doi: 10.1371/journal.pone.0336228 (PMC12668527; doi:10.1371/journal.pone.0336228)
Supplement: S6 Table — (DOCX) [file pone.0336228.s008.docx]

**S6 Table: Absorbed power (mW) in D. melanogaster tissue during the fecundity experiments for 12 locations and 2 orientations w.r.t. a 3.6 GHz dipole antenna with an input power of 23.5 dBm.**

| Location | | 1 and 2 | 3 | 4 | 5 and 6 | 7 | 8 | 9 and 10 | 11 | 12 | Mean | |
| --- | --- | --- | --- | --- | --- | --- | --- | --- | --- | --- | --- | --- |
| Orientation | ‖ | 0.261 | 0.881 | 0.215 | 0.577 | 3.28 | 0.324 | 0.454 | 1.81 | 0.317 | 0.902 | 1.91 |
|  | ꓕ | 0.420 | 3.06 | 0.248 | 1.73 | 12.3 | 1.02 | 0.966 | 5.81 | 0.657 | 2.91 |  |
