# Supplementary material for: Behaviour and reproduction of Drosophila melanogaster exposed to 3.6 GHz radio-frequency electromagnetic fields
Source: PLoS One. 2025 Dec 1;20(12):e0336228. doi: 10.1371/journal.pone.0336228 (PMC12668527; doi:10.1371/journal.pone.0336228)
Supplement: S7 Table — (DOCX) [file pone.0336228.s009.docx]

**S7 Table. ANOVA test showing how treatment interaction, treatment type and time interval contribute to the variation in the fecundity experiments, where SS = Sum of Squares, DF = Degrees of Freedom, MS = Mean Square, F = F-statistic. p < 0.05 was considered statistically significant.**

|  | **SS** | **DF** | **MS** | **F (DFn, DFd)** | **P value** |
| --- | --- | --- | --- | --- | --- |
| **Interaction** | 1.977 | 4 | 0.4944 | F (4, 50) = 1.554 | P=0.2012 |
| **Treatment** | 0.000305 | 1 | 0.0003052 | F (1, 50) = 0.0009592 | P=0.9754 |
| **Time** | 23.39 | 4 | 5.848 | F (4, 50) = 18.38 | **P<0.0001** |
| **Residual** | 15.91 | 50 | 0.3182 |  |  |
