# Supplementary material for: Behaviour and reproduction of Drosophila melanogaster exposed to 3.6 GHz radio-frequency electromagnetic fields
Source: PLoS One. 2025 Dec 1;20(12):e0336228. doi: 10.1371/journal.pone.0336228 (PMC12668527; doi:10.1371/journal.pone.0336228)
Supplement: S8 Table — (DOCX) [file pone.0336228.s010.docx]

**S8 Table. Tukey's multiple comparisons test as post-hoc analysis following significant variation with time interval found in ANOVA test. p < 0.05 was considered statistically significant**

| **Tukey's multiple comparisons test** | **Mean Diff.** | **95.00% CI of diff.** | **Adjusted P Value** |
| --- | --- | --- | --- |
| day 1-2 fecundity vs. day 3 fecundity | -0.972 | -1.624 to -0.3203 | **0.0009** |
| day 1-2 fecundity vs. day 4 fecundity | -1.438 | -2.090 to -0.7868 | **<0.0001** |
| day 1-2 fecundity vs. day 5 fecundity | -1.639 | -2.290 to -0.9869 | **<0.0001** |
| day 1-2 fecundity vs. day 6 fecundity | -1.673 | -2.325 to -1.021 | **<0.0001** |
| day 3 fecundity vs. day 4 fecundity | -0.4664 | -1.118 to 0.1852 | 0.2691 |
| day 3 fecundity vs. day 5 fecundity | -0.6666 | -1.318 to -0.01495 | **0.0426** |
| day 3 fecundity vs. day 6 fecundity | -0.7009 | -1.353 to -0.04928 | **0.0292** |
| day 4 fecundity vs. day 5 fecundity | -0.2002 | -0.8518 to 0.4515 | 0.9068 |
| day 4 fecundity vs. day 6 fecundity | -0.2345 | -0.8861 to 0.4172 | 0.8458 |
| day 5 fecundity vs. day 6 fecundity | -0.03433 | -0.6860 to 0.6173 | 0.9999 |
