# Supplementary material for: Behaviour and reproduction of Drosophila melanogaster exposed to 3.6 GHz radio-frequency electromagnetic fields
Source: PLoS One. 2025 Dec 1;20(12):e0336228. doi: 10.1371/journal.pone.0336228 (PMC12668527; doi:10.1371/journal.pone.0336228)
Supplement: S1 Fig — (DOCX) [file pone.0336228.s011.docx]

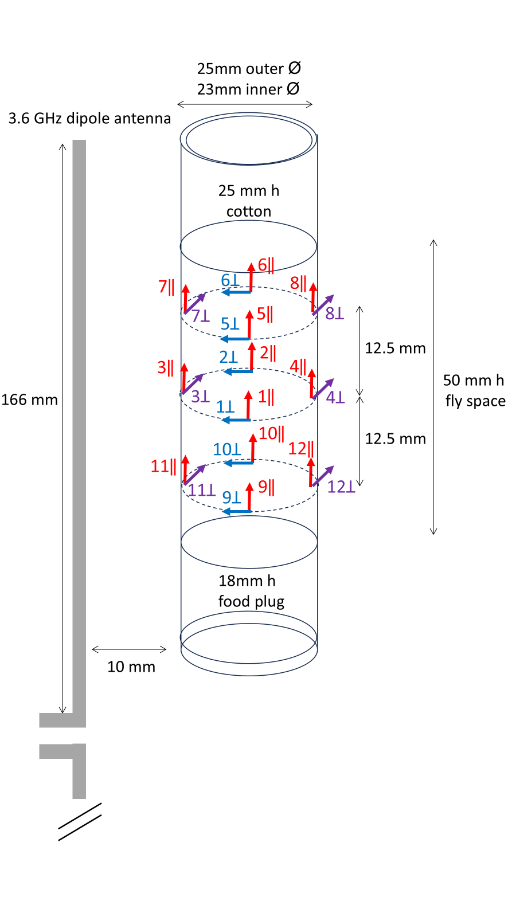


**S1 Fig. Simulation configuration of the fecundity experiments showing 12 locations of the Drosophila model each with two orientations (orthogonal ꓕ and parallel ‖ w.r.t. the dipole antenna on the left).**
