# Supplementary material for: Behaviour and reproduction of Drosophila melanogaster exposed to 3.6 GHz radio-frequency electromagnetic fields
Source: PLoS One. 2025 Dec 1;20(12):e0336228. doi: 10.1371/journal.pone.0336228 (PMC12668527; doi:10.1371/journal.pone.0336228)
Supplement: Attachment — Submitted filename: PONE-D-25-18660.pdf [file pone.0336228.s012.pdf]

PONE-D-25-18660

Behaviour and Reproduction of Drosophila Melanogaster Exposed to 3.6 GHz Radio- Frequency Electromagnetic Fields

Though some aspects of the work is well described, there is a lack of important detail when considering the experimental setup and the dosimetry, to the extent that the experiments could not be replicated and some values provided are meaningless without further information.

It should be clearly stated that exposures are to continuous wave and as such they do not represent the exposures that insects in the environment of a 5G mobile telecommunications installation would be exposed to which are characterized by high peak to average power ratios and TDD on-off slots for up and down link.

Fecundity Experiments

In this case the experiment geometry was well described, however, it would appear that the food which occupied a significant volume was not modelled nor were the dielectric properties provided. How was the exposure influenced when the flies were close to the food, how did this impact the uncertainty? The diameter of the tube was significant when compared to the spacing of the tube to the dipole antenna, the radial variation in the exposure field should be noted along with the axial variation – though it does seem that this was taken into account in the reported absorbed power levels, the link of the variation to that of the exposure field would be a nice addition. Please add information on the light dark cycles or state use for these experiments. The use of the Narda NBM (the probe type is not mentioned and should be) to measure in the near field is not appropriate due to the large averaging volume of the sensors in what is probably a 66mm diameter probe.

In the supplementary information it is stated that at 30cm from the dipole the power was 1.32nW in simulation and 1.29nW measured by the Seeed RF Explorer, without the antenna factor for this probe the results are rather meaningless, please convert to power densities i.e. W/m^2, presumably this can be done as you must have a model to have presented a simulated value.

On page 12 you talk about the volatility of the near field, do you mean variability?

Behavioral Experiments

This experiment could not be replicated as there are key details of the experiment that have not been supplied. The spacing of the antenna from the metallic grid is not stated, as this antenna radiates in both directions this is essential information. It is not made clear to the reader that the exposure is in a standing wave due to reflection from the bottom of the incubator, so this dimension is also essential to know. The standing wave is however taken into account in the extremes of the exposure over the whole exposure volume. Is there any influence on the exposure field by scattering due to the regular array of food in the exposure volume, what is its impact on the exposure uncertainty, the dielectric properties of the tube support (and diensions) and the food is not provided.

Numerical dosimetry

Though the model generated from the micro CT is very detailed with various anatomical structures identified, the dosimetry uses a model with all tissues set to the same dielectric parameters, i.e.

homogeneous, these parameters obtained from homogenized whole insects. What is the density of the tissue, this can then be used to determine measures such as average SAR. Therefore, the information in table S4 must be at the very least extremely speculative and with very high uncertainty, is its inclusion justified.
