# Supplementary material for: Behaviour and reproduction of Drosophila melanogaster exposed to 3.6 GHz radio-frequency electromagnetic fields
Source: PLoS One. 2025 Dec 1;20(12):e0336228. doi: 10.1371/journal.pone.0336228 (PMC12668527; doi:10.1371/journal.pone.0336228)
Supplement: Attachment — Submitted filename: answer to reviewers 02102025.pdf [file pone.0336228.s014.pdf]

PONE-D-25-18660

Behaviour and Reproduction of Drosophila Melanogaster Exposed to 3.6 GHz Radio-Frequency Electromagnetic Fields

Dear Editor,

Thank you for allowing us to resubmit our manuscript, providing an opportunity to address the reviewers' comments.

We are uploading our point-by-point response to your comments (below) and those made by the reviewers.

For your convenience, we have listed your comments in italic below and added our responses preceded by an asterisk (*).

We hope that these changes will persuade you to consider publishing our paper in PLOS ONE.

With kind regards,

Arno Thielens

Journal requirements:
When submitting your revision, we need you to address these additional requirements.
1. Please ensure that your manuscript meets PLOS ONE's style requirements, including those for file naming. The PLOS ONE style templates can be found at
https://journals.plos.org/plosone/s/file?id=wjVg/PLOSOne_formatting_sample_main_body.pdf
and
https://journals.plos.org/plosone/s/file?id=ba62/PLOSOne_formatting_sample_title_authors_affiliations.pdf

(*) We have adapted the manuscript according to your formatting guidelines. We did not use tracked changes for these adaptations.

2. Please note that PLOS ONE has specific guidelines on code sharing for submissions in which author-generated code underpins the findings in the manuscript. In these cases, we expect all author-generated code to be made available without restrictions upon publication of the work. Please review our guidelines at https://journals.plos.org/plosone/s/materials-and-software-sharing#loc-sharing-code and ensure that your code is shared in a way that follows best practice and facilitates reproducibility and reuse.

(*) We have reviewed the guidelines. Our data availability section lists instructions and a doi where the 3D model that was created in this paper can be found.

3. Thank you for stating the following financial disclosure:
"European Union's Horizon Europe research and innovation funding program under grant agreement No 101057216 (ETAIN)."
Please state what role the funders took in the study. If the funders had no role, please state: "The funders had no role in study design, data collection and analysis, decision to publish, or preparation of the manuscript."
If this statement is not correct you must amend it as needed.
Please include this amended Role of Funder statement in your cover letter; we will change the online submission form on your behalf.

(*) The funders had no role in study design, data collection and analysis, decision to publish, or preparation of the manuscript.

We have added this statement to the amended cover letter, and you may add it to the funding statement.

4. Thank you for stating the following in the Acknowledgments Section of your manuscript: "Part of this work is funded by the European Union's Horizon Europe research and innovation funding program under grant agreement No 101057216 (ETAIN). We also acknowledge the μ-VIS X-ray Imaging Centre (muvis.org), part of the National Facility for laboratory-based X-ray CT (nxct.ac.uk – EPSRC: EP/T02593X/1) at the University of Southampton for the provision of the μCT imaging infrastructure as well as the Insectary of the invertebrate Facility of the School of Biological Sciences of the University of Southampton for support of the behavioral studies."
We note that you have provided funding information that is currently declared in your Funding Statement. However, funding information should not appear in the Acknowledgments section or other areas of your manuscript. We will only publish funding information present in the Funding Statement section of the online submission form.
Please remove any funding-related text from the manuscript and let us know how you would like to update your Funding Statement. Currently, your Funding Statement reads as follows:
"European Union's Horizon Europe research and innovation funding program under grant agreement No 101057216 (ETAIN)."
Please include your amended statements within your cover letter; we will change the online submission form on your behalf.

(*) You can adapt the funding statement to:

"Part of this work was funded by the European Union's Horizon Europe research and innovation funding program under grant agreement No 101057216 (ETAIN). The funders had no role in study design, data collection and analysis, decision to publish, or preparation of the manuscript."

We have copied this to the revised cover letter and have removed it from the manuscript.

5. Please include captions for your Supporting Information files at the end of your manuscript, and update any in-text citations to match accordingly. Please see our Supporting Information guidelines for more information: http://journals.plos.org/plosone/s/supporting-information.

(*) We made the requested changes

If the reviewer comments include a recommendation to cite specific previously published works, please review and evaluate these publications to determine whether they are relevant and should be cited. There is no requirement to cite these works unless the editor has indicated otherwise.

(*) Not Applicable

Additional Editor Comments: The reviewers raised important criticalities in the methodology that need to be carefully addressed to make the manuscript acceptable for publication.

(*) We have addressed the methodological critiques by the reviewers and have adapted the manuscript accordingly. We are confident that the paper has improved following these adaptations and hope that it can be published in its current form.

(*) We thank the editor for reading and reviewing the paper.

PONE-D-25-18660

Behaviour and Reproduction of Drosophila Melanogaster Exposed to 3.6 GHz Radio-Frequency Electromagnetic Fields

Dear Reviewer 1,

Thank you for reading and reviewing our manuscript. We appreciate your thoughtful comments. We are providing our point-by-point response to your comments below and offer an overview of the adaptations we have made to the manuscript in response to your comments. Specifically:

- We have mentioned in the methods and results of the main text that we do have experimental exposure assessment, which can be found in the supplementary methods.
- We have clarified why positive control is not necessary in our studies and why a negative control is not necessary for behavioral experiments.
- We accounted for the use of the negative control condition used for the fecundity experiments and provide clarification on the control of the humidity during the experiment.
- We provided information on the potential effect of heating in our experiments.
- We added information on the blinding used in our experiments
- We clarified why we used a 5-5-5-day exposure schedule for the behavioral experiments and 2 days of exposure for the fecundity experiments
- We also addressed your other minor comments.

For your convenience, we have listed your comments in italic below and added our responses preceded by an asterisk (*).

We hope that these changes will persuade you to accept our paper for publication in PLOS ONE.

With kind regards,

Arno Thielens

Reviewer #1 Comments

The study analyses the impact of Drosophila melanogaster exposure to 3.6 GHz. The topic is very important given the insects' constant exposure to radiofrequency (RF) electromagnetic fields and their fundamental role in the ecosystem. The authors created a 3D model of the animal, used for numerical dosimetry, then investigated two important biological endpoints following RF exposure. The authors find no effect on locomotor activity and fecundity.

(*) Thank you for the nice summary of our work

The experiments are clearly described however the conclusions are weakened by the lack of some controls in the experimental setup.

(*) Thank you for acknowledging that our experiments are clearly described. We have provided further clarifications about the control conditions below.

The main critical issues are listed below.
Major
1) Lack of experimental dosimetry.

(*) We assume that the reviewer overlooked the Extended Methods and Extended Results sections in the Supplementary materials, where the experimental RF exposure assessment can be found. We refer the reviewer to the following Supplementary files:

- S1_File
- S2_File
- S2_Table
- S3_Table

These list the methods used for the experimental exposure assessment and the results of the exposure assessment.

In RF exposure experiments involving insects, it is currently not possible to measure internal electromagnetic fields for direct experimental dosimetry. There exist no phantoms or dielectric probes that are suitable for this species. Therefore, no other study in this field uses experimental RF dosimetry for insects. Numerical dosimetry is now the most effective method available, which is why we employed it.

We have clarified this to the readers by adding the following paragraph to the beginning of the Methods section:

“This section describes the methods used for the RF exposure experiments and the numerical dosimetry. The methods for the experimental, in-situ RF exposure assessment are described in the Extended Methods section of the S1 File.”

We also added the following sentence to the beginning of the Electromagnetic simulations section:

“There are no experimental methods available for RF dosimetry of insects. Therefore, we chose to work with numerical dosimetry in combination with experimental exposure assessment in this study. This section outlines the different steps that are taken to execute RF EM simulations of a Drosophila fly under RF-EMF exposure.”

We also added the following sentence to the beginning of the RF-EMF dosimetry Section:

“The results of the experimental RF exposure assessment can be found in the Extended Results section of the S2 File and in the S2 and S3 Tables of the Supplementary Materials. “

2) In the absence of the positive control, the validity of the methods (behavioural and fecundity experiments) cannot be confirmed.

(*) We do not find it necessary to include a positive control of these well-characterized methods and setups.

Following the first experiments monitoring *Drosophila melanogaster* circadian locomotor activity over time by Konopka and Benzer (published in PNAS 1971, https://doi.org/10.1073/pnas.68.9.2112), a commercial, standardized system was developed by Trikinetics Inc. for this purpose. Hundreds of studies have since been published, documenting its use to reliably observe locomotor activity and its responses to stimuli such as light, temperature, and vibration. We have included three example references here, where the reviewer can verify this:

1.Pfeiffenberger, C., Lear, B. C., Keegan, K. P., & Allada, R. (2010). Locomotor activity level monitoring using the Drosophila Activity Monitoring (DAM) System. Cold Spring Harbor Protocols, 2010(11), pdb-prot5518.

2. Chiu, J. C., Low, K. H., Pike, D. H., Yildirim, E., & Edery, I. (2010). Assaying locomotor activity to study circadian rhythms and sleep parameters in Drosophila. Journal of visualized experiments: JoVE, (43).

3. Green, E. W., O’Callaghan, E. K., Hansen, C. N., Bastianello, S., Bhutani, S., Vanin, S., ... & Kyriacou, C. P. (2015). Drosophila circadian rhythms in seminatural environments: Summer

afternoon component is not an artifact and requires TrpA1 channels. Proceedings of the National Academy of Sciences, 112(28), 8702-8707.

Additionally, our observations prior to the administration of the exposure (Fig 6 (a)) show that the flies exhibit circadian variations in their behavior pattern and that the system can register those.

Regarding the fecundity, it is clear that wild-type Drosophila, when left in a vial, will reproduce and that the eggs that they lay can be counted. A review of fecundity studies, such as the ones that we conducted, is provided in Chapter 6 of FlyBase Reference Report: Ashburner et al., 2005, Drosophila: a laboratory handbook. Second edition. The historical data contained there also confirm the observations we made regarding an increase in fecundity over the first few days of adulthood, which can be interpreted as validation of the assay performed. Again, numerous scientific papers utilize the same species and examine the same endpoint. A positive control to demonstrate that this happens and can be done is thus not necessary. We have added 3 references below where the reviewer can verify that this method can be used to study fecundity:

1. Barnes, A. I., Wigby, S., Boone, J. M., Partridge, L., & Chapman, T. (2008). Feeding, fecundity and lifespan in female Drosophila melanogaster. Proceedings of the Royal Society B: Biological Sciences, 275(1643), 1675-1683.
2. Huey, R. B., Wakefield, T., Crill, W. D., & Gilchrist, G. W. (1995). Within-and between-generation effects of temperature on early fecundity of Drosophila melanogaster. Heredity, 74(2), 216-223.
3. Rauser, C. L., Tierney, J. J., Gunion, S. M., Covarrubias, G. M., Mueller, L. D., & Rose, M. R. (2006). Evolution of late-life fecundity in Drosophila melanogaster. Journal of Evolutionary Biology, 19(1), 289-301.

To clarify this for readers, we have added a clarification to the methods section.

Under “Behavioral Experiments” we added:

“We did not perform a positive control for the behavioral experiments because it has been experimentally established that the activity of D. *melanogaster* can change over time and that commercially available DAM can detect this change [49]. We also did not include a negative control, as there is no known mechanism by which the presence of a non-emitting antenna could interact with the activity of D. melanogaster flies.”

[49] Pfeiffenberger, C., Lear, B. C., Keegan, K. P., & Allada, R. (2010). Locomotor activity level monitoring using the Drosophila Activity Monitoring (DAM) System. Cold Spring Harbor Protocols, 2010(11), pdb-prot5518.

Under “Fecundity Experiments” we added:

“We did not perform a positive control for the fecundity experiments because the method used here has already been experimentally established in [50].”

[50] Barnes, A. I., Wigby, S., Boone, J. M., Partridge, L., & Chapman, T. (2008). Feeding, fecundity and lifespan in female Drosophila melanogaster. Proceedings of the Royal Society B: Biological Sciences, 275(1643), 1675-1683.

3) For behavioural experiments, lack of negative control (I mean samples hosted in a standard environment/incubator to provide info on the background level of the endpoint under examination) to ensure that the exposure system does not affect the endpoint analysed.

(*) We did not include a negative control here because our sham conditions were already in a standard incubator. To the best of our knowledge, there is no mechanism by which the presence of the non-emitting exposure setup could interact with the flies. Therefore, the negative control would have the same set-up as the sham.

Additionally, we can still compare the exposed group to the sham groups to isolate the effect of RF exposure, even without a negative control.

We have added this clarification to the method section under “Behavioral experiments”:

“We also did not include a negative control, because there is no mechanism known to us by which the presence of a non-emitting antenna could interact with the activity of the D. melanogaster flies.”

4) For fecundity experiments, the sham described seems to be a negative control (see above) rather than a sham. In addition, it seems that temperature is controlled but not the humidity of the room.

Any difference between sham and negative control conditions would have to be attributable to the impact of a non-emitting RF source-connected antenna. Again, there are no mechanisms known to us by which this would manifest. Indeed, the reviewer is right to note that we included a sham control for the locomotor assay and a negative control for the fecundity assay; however, no effect relative to the 5G exposure was observed in either scenario. Humidity was controlled from day 2 onwards by co-housing experimental and control vials in the same humidified box. We added this to the methods section.

5) The authors should clarify whether the exposure conditions cause heating of the sample or not.

(*) It is hard to determine the temperature of the individual flies. For this reason, the behavioral experiment was designed this way. Suppose there had been significant heating (more than a couple of degrees). In that case, it should have been evident in the locomotor patterns, since it is known that temperature affects the locomotor behavior of D. melanogaster [51]. However, we did not observe that, so we do not think there was significant heating.

Similarly, experimental evidence has shown that temperature also affects Drosophila fecundity [52, 53]. Since we did not find any effect on the fecundity, we also hypothesize that there was no significant heating during the fecundity experiments.

[51] Tomioka, K., Sakamoto, M., Harui, Y., Matsumoto, N., & Matsumoto, A. (1998). Light and temperature cooperate to regulate the circadian locomotor rhythm of wild type and period mutants of Drosophila melanogaster. *Journal of insect physiology*, *44*(7-8), 587-596.

[52] Gandara, A. C. P., & Drummond-Barbosa, D. (2022). Warm and cold temperatures have distinct germline stem cell lineage effects during Drosophila oogenesis. Development, 149(5), dev200149.

[53] Huey, R. B., Wakefield, T., Crill, W. D., & Gilchrist, G. W. (1995). Within-and between-generation effects of temperature on early fecundity of Drosophila melanogaster. Heredity, 74(2), 216-223.

Nonetheless, we do acknowledge that if one were able to measure the temperature of the individual flies during the experiments, this would improve the experiment.

We have added the following information to the “Results and discussion” section:

Under “Behavioral experiments”

“We controlled the environmental temperature but did not monitor the temperature of the individual flies in our experiment, as it would have been nearly impossible to do so Tomioko et al. [51] demonstrated that temperature influences the circadian locomotor rhythm of D. melanogaster. Since we did not observe any significant changes in locomotor activity due to RF exposure, we hypothesize that there was no significant heating. “

Under “Fecundity experiments”

“While we controlled the environmental temperature, we did not monitor the temperature of the individual flies during the RF exposure. Hence we cannot exclude the possibility of

heating occurring. However, Ganadara et al [52] and Huy et al. [53] demonstrated that temperature has an influence on D melanogaster fertility. Since we did not observe any effect, we hypothesize that there was no significant heating in our experiments. "

6) The authors should state whether the experiments are conducted blindly.

(*) During the behavioral experiments, the flies had no way of knowing whether the RF exposure was on or off, as the RF generator was located outside the climate chamber. The locomotor data is analyzed automatically and objectively according to a prespecified algorithm. Therefore, the investigator who receives the results for the final interpretation is not blinded to the exposure conditions, but cannot influence data collection or analytical approach.

For the Fecundity data, the vials were coded, and eggs were counted without immediate knowledge of the exposure conditions. Decoding and unblinding were done during statistical analyses. The same investigator performed the coding and decoding. The investigator cannot influence the number of eggs produced by the flies. The flies were again unable to determine whether the antenna in the exposure experiment was emitting RF exposure or not, since the source was not within their field of view. However, they can observe the presence of the antenna.

We added this information to the paper in the "Methods" section.

Under "Behavioral experiments":

"The flies were blind to the RF exposure as they had no way of knowing whether the RF source outside of the chamber was on or off. The data activity recording and analysis are performed automatically by the DAM device and software, which are insensitive to RF exposure conditions. Therefore, the researcher who received the data and analysis was not blind to the exposure but could also not influence the results."

Under "Fecundity experiments":

"The flies in the exposure experiment could see the antenna but could not see the RF source and thus could not know whether the antenna was emitting RF fields or not. The vials were coded, and eggs were counted without immediate knowledge of the associated treatment. Decoding was performed during statistical analyses by the same investigator who had coded

the data. The investigator cannot influence the number of eggs produced by the flies."

Minor

For behavioural experiments
1) The choice of exposure schedule (5 days before, 5 days of exposure, 5 days after) should be justified.

(*) This design is a compromise between maintaining good survival throughout the experiment and having a sufficient number of days available for each segment to determine reproducible periodicity. We added this to the "Methods" section under "Behavioral experiments":

"The 5-5-5 days design was used as a compromise between maintaining good survival throughout the experiment and having a sufficient number of days available for each segment to determine reproducible periodicity"

2) The authors claim to use 32 flies for the experiments (line 11 of page 8) but in Figure 6, 9 exposed males are shown, and in the caption of the same figure, 12 exposed males are mentioned. Please clarify this inconsistency.

(*) Flies that were considered to have died during the experiment due to persistent immobility were excluded.

3) The aim of the paper is to analyse the possible effect of RF exposure so in my opinion the comparison of interest, to be reported in the main text rather than in the supplementary, is RF vs sham and not those reported in Figure 6 (pre RF vs RF, RF vs post RF and pre RF vs post RF).

(*) Following this request, we moved Table S7 (now Table I) to the main paper and added the following paragraph to the main paper (Results and discussion, Behavioral experiments):

"Table 1 also lists the comparative testing of the sham-exposed versus RF-exposed flies, separated for male and female flies. We found significant differences in activity counts for female flies and in the periodicity for male flies using the Kruskal-Wallis test at $p<0.05$ for the entire pre-sham-post sequence versus the entire pre-5G-post sequence. Post-hoc

testing of the paired pre-treatment, the sham-exposure versus the Rf-exposure, and the post-treatment data showed no significant differences."

For fecundity experiments<br>*4) Please provide rationale for the duration of the exposure (2 days).*

(*) This exposure scheme allowed for the analysis of both immediate and indirect effects on fecundity. Additionally, we would rather err on the side of saturating exposure to detect an effect rather than miss an effect requiring sustained exposure.

We added this to the "Methods" section under "Fecundity experiments":

"Working with 48h of exposure allowed for the analysis of both immediate and indirect potential effects of the exposure"

5) Clarify whether the exposure is continuous or intermittent.

(*) The exposure was continuous for the 2 days in the fecundity experiments and continuous for the indicated 12-hour periods in the five 12-hour on:12-hour off exposure cycles of the locomotor assays. We added this info the "Methods section", under "Behavioral experiments":

"After 5 days, the second 5-day interval started in which experimental flies were exposed to 12h on (from 09:00 to 21:00) /12h off RF-EMF (**continuous exposure**) irradiation cycles."

And "Fecundity experiments":

"After 48 hours of **continuous RF-EMF** or negative control, flies from each vial were transferred to a fresh vial. "

We also added to the "Results and discussion" section:

Under "Fecundity experiments":

"In the fecundity and behavioral experiments**, we used continuous RF-EMF exposure**."

(*) We thank the reviewer for their careful reading and review of the paper.

PONE-D-25-18660

Behaviour and Reproduction of Drosophila Melanogaster Exposed to 3.6 GHz Radio-Frequency Electromagnetic Fields

Dear Reviewer 2,

Thank you for reading and reviewing our manuscript. We appreciate your thoughtful comments. We are providing our point-by-point response to your comments below and offer an overview of the adaptations we have made to the manuscript in response to your comments. Specifically, we have:

- We clarified that we used continuous RF exposure and that real telecommunication networks use modulated signals.
- We added the reviewer's important observation that the absorbed power in the flies depends on the separation distance to the antenna.
- We added more information on the simulations and measurements.

For your convenience, we have listed your comments in italic below and added our responses preceded by an asterisk (*).

We hope that these changes will persuade you to accept our paper for publication in PLOS ONE.

With kind regards,

Arno Thielens

Reviewer #2: Though some aspects of the work is well described, there is a lack of important detail when considering the experimental setup and the dosimetry, to the extent that the experiments could not be replicated and some values provided are meaningless without further information. The addition of the missing information could improve the paper.

(*) We have added the requested information.

It should be clearly stated that exposures are to continuous wave and as such they do not represent the exposures that insects in the environment of a 5G mobile telecommunications installation would be exposed to which are characterized by high peak to average power ratios and TDD on-off slots for up and down link.

(*) Following your comments and those of Reviewer 1, we clarified in the methods section that we used continuous exposure.

Under "Behavioral experiments"

"After 5 days, the second 5-day interval started in which experimental flies were exposed to 12h on (from 09:00 to 21:00) /12h off RF-EMF (**continuous exposure**) irradiation cycles."

And "Fecundity experiments":

"After 48 hours of **continuous RF-EMF** or negative control, flies from each vial were transferred to a fresh vial. "

We have also added to the "Results and discussion" section that real telecommunication networks use signal modulation and do not employ pure continuous wave emissions.

Under "Fecundity experiments":

"In the fecundity and behavioral experiments, **we used continuous RF-EMF exposure**, while real telecommunication networks use modulated signals to transmit information. In our follow-up studies, we will use modulated signals to fully emulate RF-EMF exposure in telecommunication networks."

Fecundity Experiments
In this case the experiment geometry was well described, however, it would appear that the food which occupied a significant volume was not modelled nor were the dielectric properties provided.

(*) The food was indeed not modelled here (see next answer). We have added this to the text of the extended Methods:

“Mann-Whitney U testing on measurements with and without vials could not prove that the vials attenuate the electromagnetic field. Therefore, the vials were not considered in the near-field simulations. The food within the vials was also not included in the simulations.”

How was the exposure influenced when the flies were close to the food, how did this impact the uncertainty?

(*) As stated above, we did not model the presence of the food. However, it is essential to note that adults are mostly found on the walls of the tubes. This is why we chose to model the exposure by placing the Drosophila model on the virtual walls of the tubes, as illustrated in the S1 Figure.

The diameter of the tube was significant when compared to the spacing of the tube to the dipole antenna, the radial variation in the exposure field should be noted along with the axial variation – though it does seem that this was taken into account in the reported absorbed power levels, the link of the variation to that of the exposure field would be a nice addition.

(*) We took the radial variation into account, as shown in the S1 Figure, which describes the simulation configuration. As the S6 Table shows, the absorption values at the locations close to the dipole antenna (3,7, 11) result in higher absorption values than those at the other side of the tube (4,8,12). We have added this observation by the reviewer to the results section:

“The absorption at the locations close (11 mm) to the antenna (3,7,11) of 0.881-12.3 mW is higher than the one at the opposite side of the tube (locations 4,8,12) at 34 mm from the antenna, 0.215-1.02 mW for an emitted power of 23.5 dBm at 3,6 GHz. Separation distance from the antenna is thus an important factor in this variation. “

Please add information on the light dark cycles or state use for these experiments.

(*) The fecundity experiments were conducted in a 12-hour light:12-hour dark environment illuminated by fluorescent tube lights. We added this to the Methods section in the appropriate subsection:

“The setup was placed in a climate-controlled room, as shown in Figs 3 (b) and (c), in a 12-hour light:12-hour dark environment illuminated by fluorescent tube lights. Control flies (negative) were kept in a separate room under identical temperature and lighting conditions for the first two days. “

The use of the Narda NBM (the probe type is not mentioned and should be) to measure in the near field is not appropriate due to the large averaging volume of the sensors in what is probably a 66mm diameter probe.

(*) The EF 0691 E-Field Probe was used in combination with the Narda NBM. We have added this to the text of the Extended Methods, as requested by the reviewer:

"…and a Narda NBM Probe with EF 0691 E-Field probe (Narda, Hauppage, NY) was used to register root-mean-square electric field strength 100 kHz- 6 GHz every 2 seconds. "

We do not agree that the values measured in the near field by the probe are meaningless. They clearly demonstrate that the RF fields originate from the antenna and not from any other source, which was the primary purpose of including those measurements. The field used for dosimetry is measured in the far field of the antenna, where the probe is accurate.

In the supplementary information it is stated that at 30cm from the dipole the power was 1.32nW in simulation and 1.29nW measured by the Seeed RF Explorer, without the antenna factor for this probe the results are rather meaningless, please convert to power densities i.e. W/m2, presumably this can be done as you must have a model to have presented a simulated value.

(*) Unfortunately, the RF explorer does not provide power density values. Nonetheless, the power registered by the device scales linearly with the power density if it were multiplied by the device's unknown antenna factor. Therefore, the values listed in Table S3 can still be used to demonstrate that the measured power density with and without vials was statistically not different from each other, and that we could therefore omit the vials from the modeling.

Because we did not measure power densities and the device also does not provide them, we prefer to list what we actually measured.

The simulated antenna aperture is around $2.9\ 10^{-8}\ m^2$.

On page 12 you talk about the volatility of the near field, do you mean variability?

(*) Correct, we changed this in the text. Thank you for pointing this out.

Behavioral Experiments
This experiment could not be replicated as there are key details of the experiment that have not been supplied.

(*) We have provided the information you requested below and in the paper.

The spacing of the antenna from the metallic grid is not stated, as this antenna radiates in both directions this is essential information.

(*) We added the spacing of the antenna to the metal grid (6.4cm) to the text.

"A directive RF-EMF-emitting antenna (Taoglass Maximus Flexible Ultra-Wide-Band Antenna, 700–6000 MHz) was positioned 25 cm above the DAM device, exceeding the far-field limit at 3.6 GHz, and suspended 6.4 cm under a metal grid."

It is not made clear to the reader that the exposure is in a standing wave due to reflection from the bottom of the incubator, so this dimension is also essential to know. The standing wave is however taken into account in the extremes of the exposure over the whole exposure volume.

(*) We believe that reflections from the bottom are minimal here due to the water on the bottom, but we have not modeled it, indeed. Therefore, we do not make assumptions on the angle of incidence in the dosimetry of the behavioral experiments

Our measurements were taken in situ, so they will account for any reflections that may exist.

We added this to the "Results and discussion" under "Absorbed power in D. melanogaster during the behavioral experiments ":

"In our simulations, we did not consider standing waves that could occur due to interference between reflections within the incubator. However, we do take variation in absorption into account by considering a range of potential angles of incidence."

Is there any influence on the exposure field by scattering due to the regular array of food in the exposure volume, what is its impact on the exposure uncertainty, the dielectric properties of the tube support (and diensions) and the food is not provided.

(*) We did not model the food, the tube, the DAM device, etc. for the behavioral experiments, we used far-field plane wave simulations. We added this to the "Results and discussion" under "Absorbed power in D. melanogaster during the behavioral experiments ":

"We also did not model the presence of the food, the DAM, and the tubes, and only considered plane wave exposure of the fly model in free space."

Numerical dosimetry
Though the model generated from the micro CT is very detailed with various anatomical structures identified, the dosimetry uses a model with all tissues set to the same dielectric parameters, i.e. homogeneous, these parameters obtained from homogenized whole insects.
What is the density of the tissue, this can then be used to determine measures such as average SAR.

(*) Unfortunately, we do not have density values at this time and can therefore not calculate SAR values.

Therefore, the information in table S4 must be at the very least extremely speculative and with very high uncertainty, is its inclusion justified.

(*) We believe it is worthwhile to present the results due to the methodological novelty of being able to calculate absorbed powers at the tissue level in insects.

We added this to the “RF-EMF dosimetry” section of the “Results and discussion”:

“The accuracy of the tissue-specific values can be improved by using tissue-specific dielectric properties, which currently do not exist for this insect. However, the values demonstrate the model's usability in calculating such values.”

(*) We thank the reviewer for their careful reading and review of the paper.
